# Herbo-mineral formulation ‘Ashwashila’ attenuates rheumatoid arthritis symptoms in collagen-antibody-induced arthritis (CAIA) mice model

**DOI:** 10.1038/s41598-019-44485-9

**Published:** 2019-05-29

**Authors:** Acharya Balkrishna, Sachin Shridhar Sakat, Kheemraj Joshi, Sandeep Paudel, Deepika Joshi, Kamal Joshi, Ravikant Ranjan, Abhishek Gupta, Kunal Bhattacharya, Anurag Varshney

**Affiliations:** 1 Drug Discovery and Development Division, Patanjali Research Institute, NH-58, Haridwar, 249 405 Uttarakhand India; 2 University of Patanjali, Patanjali Yog Peeth, Roorkee-Haridwar Road, Haridwar, 249 405 Uttarakhand India; 3 Center for Nanotechnology and Nanotoxicology, Harvard T.H. Chan School of Public Health, 665 Huntington Avenue, Boston, 02115 MA United States of America

**Keywords:** Rheumatoid arthritis, Rheumatoid arthritis

## Abstract

Rheumatoid arthritis (RA) is a chronic inflammatory autoimmune disorder that affects joints of hands and feet and introduces injury in secondary organs such as cardiac tissue. In the present study, we induced RA in male Balb/c mice (CAIA) using collagen-antibody cocktail (C-Ab) and lipopolysaccharide intraperitoneal injections. Induction of RA in the animals was detected through the loss of body weight, food, and water consumption, pedal edema, increased arthritis score of the paw and ankle, increase in radiological and histological lesion score of ankle and knee joints and enhanced pain perception in the C-Ab induced RA animals. Ashwashila is a herbo-mineral medicine from Indian Ayurvedic system. Human equivalent doses of Ashwashila (ASHW) and standard of care, Methotrexate were given to the CAIA animals for two weeks. ASHW treatment significantly reversed the effect of C-Ab with reduced pedal edema, arthritis score, radiological and histological lesion scores in ankle-joint, knee-joint and articular cartilage, reduced pain perception. These effects were comparable with the Methotrexate treatment. In human monocytic (THP-1) cells, ASHW was found to be biocompatible at *in-vitro* test doses. The anti-arthritis mechanism of action for ASHW was established through the suppression of pro-inflammatory cytokines such as IL-1β, IL-6, TNF-α; and upstream regulator, NF-κB. Taken together, we show the pre-clinical efficacy of ASHW in reducing RA associated symptoms by controlling inflammation and suggest it as a potential therapeutic candidate for rheumatoid arthritis.

## Introduction

Rheumatoid arthritis (RA) is a chronic inflammatory autoimmune disease that affects between 0.5–1% population of the world with women representing the majority of the diseased population^1^. Several factors such as genetic, epigenetic, gender, environment and lifestyles play a crucial role as precursors for this disease. RA is characterized by local and systemic inflammation mostly occurring in the joints through the development of auto-antibodies to immunoglobulin G (IgG) such as rheumatoid factor (RF) and citrullinated proteins^1^. Under chronic and untreated conditions, RA can lead to severe and irreversible damage of the joints through inflammation, edema, cartilage and bone damages.

The clinical symptoms of the RA disease are the soft synovial swelling along with morning stiffness and tenderness of metacarpophalangeal and proximal interphalangeal joints of the hands and feet, along with wrist, ankle, elbow, shoulder, knee and hip joints^2^. Synovial region of the joint is the primary location for detectable RA. Pathogenic changes induced in the synovial area are mainly, increase and activation of synoviocytes A (macrophages) and B (fibroblast-like). Increased synoviocyte activity leads to the release of pro-inflammatory cytokines, chemokines and collagen-degrading proteases such as matrix metalloproteinases; and small molecule cell-signaling mediators such as prostaglandins and leukotrienes^3–5^. Other changes associated with the development of RA are infiltration of immune cells such as CD4+ memory T cells, B cells, plasmablasts, and plasma cells into the synovial sub-lining, producing RF and citrullinated proteins^1^. The cardinal signs of RA are damage to the articular cartilage and bone, along with visible pannus formation in the joints. Treatment of RA includes non-steroidal anti-inflammatory drugs (NSAIDs), disease modifying anti-rheumatic drugs (DMARDs), tumor necrosis factor alpha (TNF-α) inhibitors, IL-6 inhibitors, T-cell activation inhibitors, B-cell depletors, Kinase (JAK) inhibitors, immune-suppressants, and steroids. Other than these medications, appropriate changes in lifestyle such as regular exercise are also advisable.

Collagen type II is the major component of the joint’s cartilage matrix protein. The RA in animals models are induced by systemic administration of a cocktail of monoclonal antibodies (C-Ab) that target the various regions of collagen type II. Animals are further stimulated by lipopolysaccharide (LPS) for induction of joint inflammation^6^. Pathogenic features of collagen type II antibody induced RA in animal models include elevated arthritic scores, pedal edema, synovitis with infiltration of polymorphonuclear and mononuclear cells, pannus formation, collagen degradation, and bone erosion^7^.

‘Ashwashila’ (ASHW) is a herbo-mineral formulation containing aqueous extract of ‘Ashwagandha’ (*Withania somnifera*, family: Solanaceae) commonly known as ‘Indian Winter Cherry’ or ‘Indian Ginseng’; and dry powder of ‘Shilajit’ found as a blackish-brown exudate present on the rocks of the Himalayas between Arunachal Pradesh and Kashmir, in India^8^. Under severe RA conditions in animals, ‘Ashwagandha’ herbal extracts have been found to reduce inflammatory responses^9,10^. ‘Ashwagandha’ herbal extracts have shown that its withanolides components modulate proliferation of breast cancer tissue through induction of FOXO3a protein and pro-apoptotic protein BIM, leading to induction of apoptosis in breast cancer cells^11^. Similarly, ‘Withaferin-A’, a component of the ‘Ashwagandha’ has been reported to bind with the cysteine residues of the IKK-β kinase. This deactivation of the IKK-β kinase exerts anti-inflammatory response by blocking of downstream NFκβ activation^12^. The second component of ASHW, ‘Shilajit’ is formed from gradual decomposition of plant matter contains both humic and non-humic constituents^8^. ‘Shilajit’ has been used extensively in ancient herbal formulations as a rejuvenator and anti-aging agent. Fulvic acid present in ‘Shilajit’ has been found to have immunomodulatory and psychoactive behavior^13^. Treatment of ‘Shilajit’ prevents self-aggregation of tau fibrils, that is responsible for the development of Alzheimer’s disease^14,15^. ‘Shilajit’ also contains elemental Selenium that a has a significant anti-inflammatory function, as an inhibitor for COX-2 and TNF-α activity^16,17^. Dietary supplement of Selenium also decreases mechanically induced osteoarthritis; and increases levels of anti-oxidative enzymes in the knee joints^18^.

Combined treatment with herbal extracts of both ‘Ashwagandha’ and ‘Shilajit’ has been found to work as a nootropic or psychoactive drug, reducing addiction to alcohol consumption in the Swiss albino mice^19^. Both ‘Ashwagandha’ and ‘Shilajit’ are present in the ASHW herbal formulation in equal quantity. However, no study has been reported to date to determine the efficacy of ASHW on RA and inflammation.

In the present study, the efficacy of ASHW herbo-mineral formulation in reducing the inflammatory response to RA in the joints of Balb/c mice has been tested. RA was induced in the Balb/c mice using a collagen-antibody cocktail (C-Ab) and lipopolysaccharide (LPS). The collagen antibody-induced arthritis (CAIA) animals were treated with ASHW and Methotrexate (MTX), as the reference standard of care for two weeks. These animals were studied for their feeding and water intake habits, body weight changes along with modifications in the symptoms for arthritic edema, pain perception, radiological and histopathological analysis of the ankle and knee joints. For determining the mechanism of action, we treated the LPS stimulated human monocytic (THP-1) cells with ASHW and studied the release of interleukin one beta (IL-1β), IL-6 and Tumor Necrosis Factor-alpha (TNF-α); and expression of upstream regulatory protein, NFκB. Our results showed that the ASHW exhibited promising efficacy in reducing the RA symptoms in the diseased animals through the modulation of cell-signaling components associated with inflammation.

## Results

**Figure 1:**
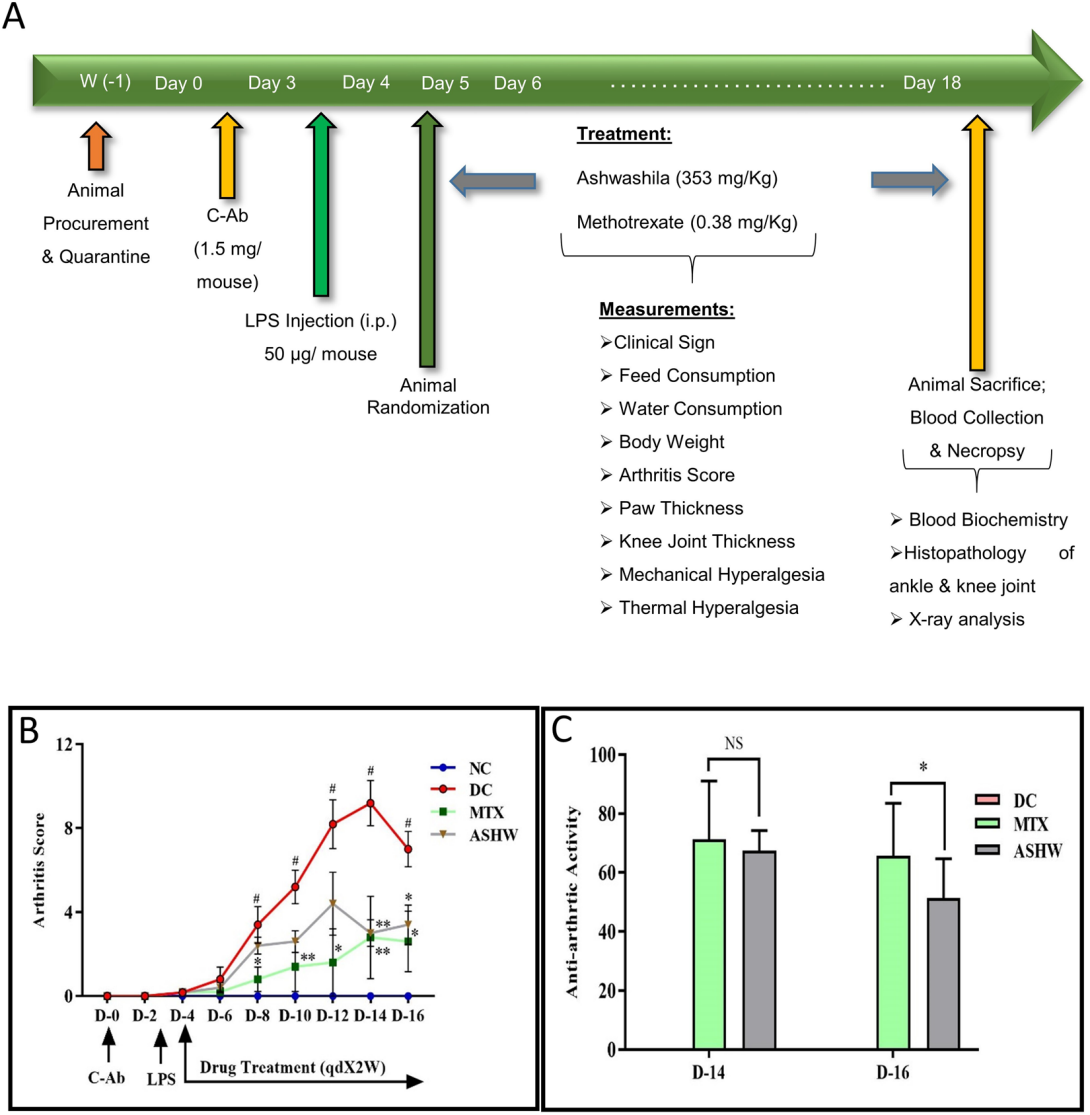
Study Design and Modulation of Arthritis Score by Ashwashila. (**A**) Male Balb/c mice of 6–8 weeks’ age were injected intraperitoneally with 1.5 mg/mouse dose of anti-collagen antibody (C-Ab) cocktail and 50 µg/mouse of bacterial lipopolysaccharide (LPS). All the disease and treatment group animals were selected randomly from the C-ab induced arthritis (CAIA) animals. The normal (NC) and disease control (DC) animals were treated with sodium carboxymethyl cellulose (Na-CMC) while the treatment group CAIA mice were treated with 353 mg/kg oral dose of ASHW and 0.38 mg/kg oral dose of MTX given every alternate day for two weeks. Physical and clinical parameters of the animals were measured every day throughout the experimental duration. (**B**) Arthritis score showed an increase in the CAIA animals indicating the onset of RA. Treatment of the CAIA mice with ASHW and MTX significantly reduced the arthritis score indicating a reduction in pedal swellings. (**C**) Anti-arthritic activity analysis based on the arthritis score showed similar efficacy for both ASHW and MTX except on day 16. Results represent Mean ± SEM. A one-way analysis of variance (ANOVA) followed by Dunnett’s multiple comparison t-test was used to calculate the statistical difference. Student unpaired t-test was used to calculate the statistical difference in comparison to MTX (p-value * ≤ 0.05; ** ≤ 0.01).

In the present study, disease control (DC) animal model, the collagen antibody-induced arthritis (CAIA) Balb/c mice, showed severe induction of rheumatoid arthritis (RA) and associated distress through rapid reduction in their body weight, feed, and water intake habits (Fig. 1A, Suppl. Fig. 1A–C). Treatment of the CAIA animals with 353 mg/kg dose of ASHW (mice equivalent human dose of 2000 mg/day) every day for two weeks did not affect the induced loss of body weight along with the reduced food and water consumption quantities (Suppl. Fig. 1A–C). Treatment of the CAIA animals with 0.38 mg/kg dose of standard of care drug, MTX every alternate day for two weeks showed significant recovery of body weight, as compared to the DC animals (Suppl. Fig. 1A–C), without any observable changes in the reduced food and water consumption habits.

The onset of RA in the DC animals following C-Ab and LPS treatments was also visible through a constant increase in arthritis score compared to the normal control (NC) animals (Fig. 1B). Treatment of the CAIA animals with the 353 mg/kg dose of ASHW or with 0.38 mg/kg dose of MTX induced a significant reduction in the arthritis score till the end of the study period (Fig. 1B). Statistically significant reduction of arthritis score in ASHW-treated diseased animals was found on days 14 (p-value ≤ 0.01) and 16 (p-value ≤ 0.05); and for reference drug MTX on days 8 (p-value ≤ 0.05), 10 (p-value ≤ 0.01), 12 (p-value ≤ 0.05), 14 (p-value ≤ 0.01) and 16 (p-value ≤ 0.05), when compared to the DC animals. The anti-arthritic activities shown by ASHW and reference drug MTX were found to be statistically indistinguishable on day 14 (Fig. 1C).

**Figure 2:**
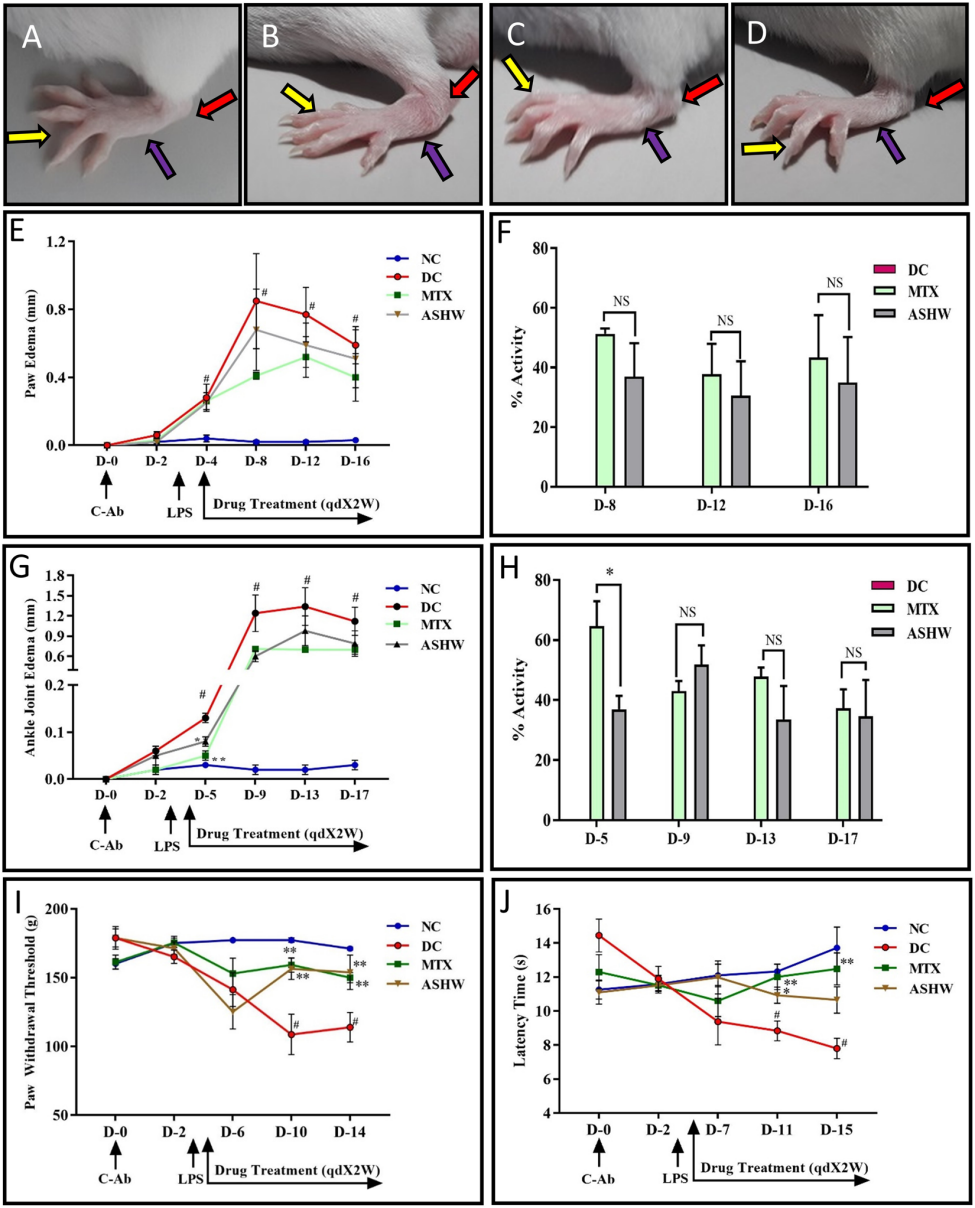
Paw and Ankle Edema Modification by Ashwashila. (**A**) Normal control (NC) Balb/c mice showing digits (yellow arrow), foot (blue arrow) and ankle (red arrow). (**B**) Development of digits, foot and ankle edema in collagen antibody-induced arthritis (CAIA) disease control (DC) animal. (**C**) Reduction in the inflammation of digits, foot and ankle edema in CAIA mice treated with 353 mg/kg dose of Ashwashila (ASHW) every day for two weeks. (**D**) Reduction in inflammation of digits, foot and ankle edema in CAIA mice treated with 0.38 mg/kg dose of Methotrexate (MTX) every alternate day for two weeks (MTX). (**E**) Increase in paw edema of the observed in the CAIA animals. Treatment of the CAIA mice with ASHW or MTX induced significant reduction in the paw edema. (**F**) Percentage (%) activity of the ASHW or MTX treatments in reducing paw edema in the CAIA animals indicated similar efficacy. (**G**) Increase in ankle-joint edema was observed in the CAIA animals. Treatment with ASHW or MTX significantly reduced the ankle-joint edema in the CAIA animals. (**H**) Percentage (%) activity of the ASHW or MTX treatments in reducing knee-joint edema in the CAIA animals indicated similar efficacy. (**I**) Paw withdrawal threshold was measured using Randall Selitto (Mechanical hyperalgesia) parameter. The results showed a significant increase in the paw withdrawal threshold in the CAIA animals. Increase in mechanical hyperalgesia was recovered in the CAIA animals treated with Ashwashila (ASHW) and Methotrexate (MTX). (**J**) Thermal hyperalgesia test showed reduced in the latency time of CAIA animals followed by significant recovery when treated with ASHW and MTX. Values in the results are Mean ± SEM. A one-way analysis of variance (ANOVA) followed by Dunnett’s multiple comparison t-test was used to calculate the statistical difference. Student unpaired t-test was used to calculate the statistical difference in comparison to MTX (p-value # ≤ 0.05; * ≤ 0.05).

According to the American College of Rheumatology (ACR) and European League Against Rheumatism (EULAR) on RA classification, swelling of at least one joint is a requisite criterion^20^. In our study, edema examination of the paw and ankle joints showed increased swelling of the ankle, feet, and digits in the CAIA animals as compared to the NC animals (Fig. 2A,B). Treatment of the CAIA animals with herbal formulation ASHW and reference drug MTX prompted a reduction in the swelling in the ankle, feet, and digits (Fig. 2C,D). Visual observation of edema was further complemented by measuring the paw and ankle thickness in the animals (Fig. 2E,G). The DC animals showed significant (p-value ≤ 0.05) increase in the paw and ankle thickness (Fig. 2E,G). Treatment of the CAIA animals with ASHW (paw edema - Day 8: 37.0 ± 11.2%; Day 12: 30.5 ± 11.6%; Day 16: 34.9 ± 15.3%) and MTX showed a considerable decrease in paw and ankle edema measured at different days (Fig. 2E,G). Those observed reduction in paw edema following drug treatments were statistically not significant. However, reduction in the ankle edema on day five was statistically significant for both the ASHW (p-value ≤ 0.05) and MTX (p-value ≤ 0.01) treatments (Fig. 2G). ASHW showed equipotent efficacy in controlling pedal and ankle joint edema in comparison to the reference care drug MTX throughout the study period, except on day 5, wherein MTX displayed superior effects (p-value ≤ 0.05) in controlling ankle joint edema (Fig. 2F,H).

During the onset of RA, there is a significant induction of neuroinflammation and hyperalgesia, due to the release of pro-inflammatory cytokines and chemokines^21^. Clinical evidence for enhanced hyperalgesia has been demonstrated in RA patients^22^. Pain perception analysis of the CAIA animals was performed using Randall-Selitto test (Mechanical Hyperalgesia) and hot plate test (Thermal Hyperalgesia), on different days of treatments (Figs 1A and 2I,J). Treatment of the CAIA animals with ASHW showed noticeable reduction (p-value ≤ 0.01) in their pain sensitization towards Randall-Selitto test at days 10 and 14 (Fig. 2I). Similarly, treatment of the CAIA animals with the reference drug MTX decreased their pain sensitivity (p-value ≤ 0.01) at days 10 and 14 (Fig. 2I). Hot plate test further confirmed the reduction of pain sensitivity following ASHW or MTX treatments in the CAIA animals (Fig. 2J). Statistically significant decrease in the pain sensitivity of the CAIA animals tested by hot plate test was observed in ASHW treatment on day 11 (p-value ≤ 0.05) and for MTX treatment on days 11 (p-value ≤ 0.01) and 15 (p-value ≤ 0.01) (Fig. 2J).

**Figure 3:**
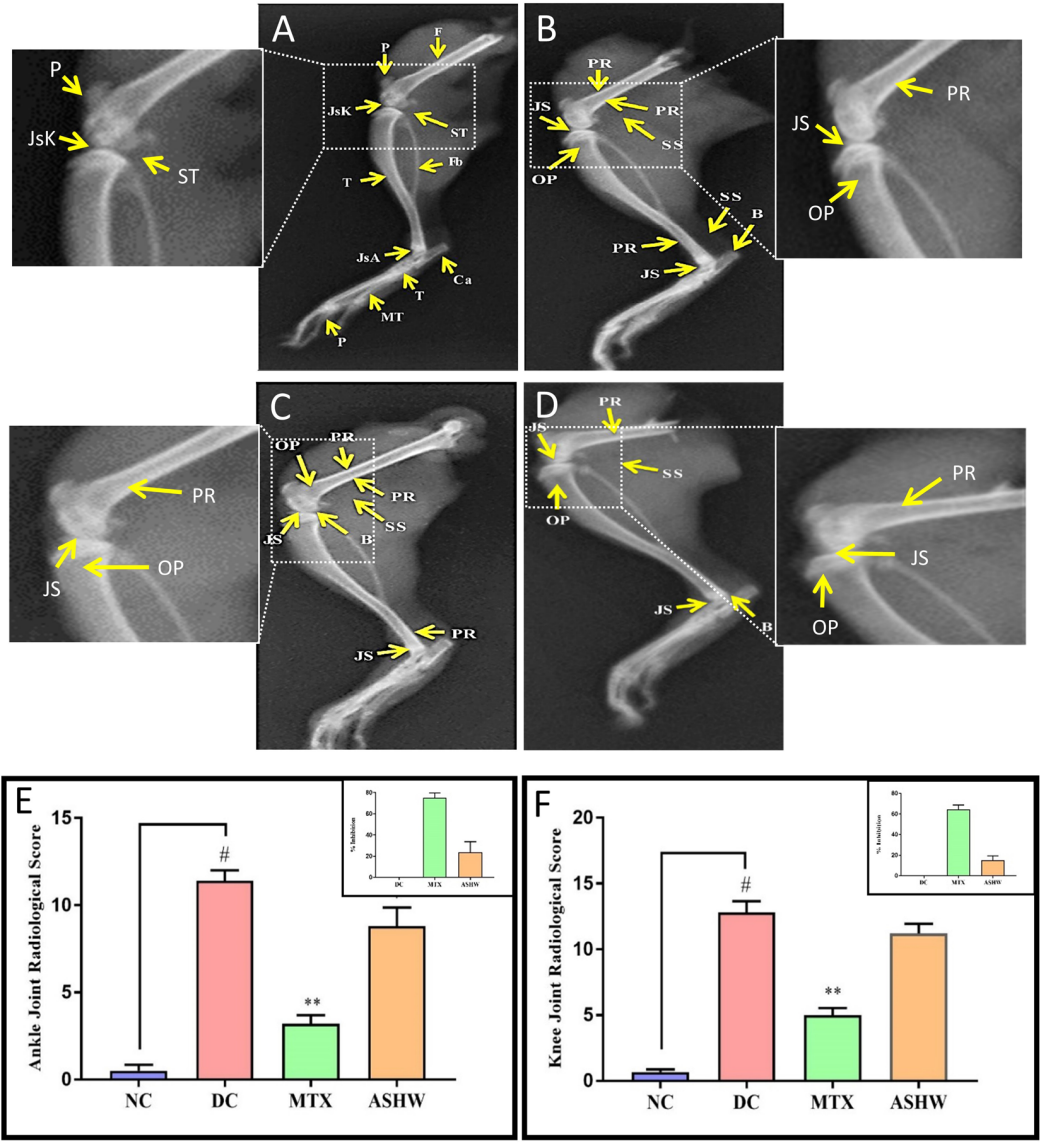
Radiological Analysis of Rheumatoid Arthritis (RA) Diseased and ASHW Treated Mice. X-ray analysis of (**A**) Normal control (NC) animal pedal region radiological analysis showing Tibia (T), Fibula (Fb), Calcaneum (Ca), Patella (P), Femur (F), Tarsals (T), Metatarsals (MT) and Phalanges (P). Normal joint space with healthy cartilage at knee joint (JsK) and ankle joint (JsA) and periarticular soft tissue (ST). Inset: Magnified normal knee-joint region of NC. (**B**) Disease control (DC) animal showing periosteal reaction/hypertrophy (PR), bone erosion (B), soft tissue swelling (SS), narrowed joint space (JS) and osteoporosis (OP). Inset: Magnified knee-joint region of the DC animal showing PR, JS and OP. (**C**) Methotrexate (MTX) treated CAIA animal showing PR, B, SS, JS, and OP. Inset: Magnified knee-joint region of MTX treated animal showing PR, JS, and OP. (**D)** Ashwashila (ASHW) treated CAIA animal showing PR, B, SS, JS, and OP. Inset: Magnified knee-joint region of the ASHW treated animal showing PR, JS, and OP. (**E**) Ankle radiological score showed significant damage in the DC animals as compared to the NC animals, and reduced ankle radiological score was following treatment with ASHW or MTX. (**F**) The knee-joint radiological score showed increased damage in the DC animals at the onset of RA disease and marginal reduction following treatment with ASHW or MTX. Values in the results are Mean ± SEM. A one-way analysis of variance (ANOVA) followed by Dunnett’s multiple comparison t-test was used to calculate the statistical difference. Student unpaired t-test was used to calculate statistical difference in comparison to MTX (p-value # ≤ 0.05; * ≤ 0.05; ** ≤ 0.01).

The severity of the C-Ab induced RA disease in the animals was studied using X-ray techniques, and radiological scoring was enumerated, considering different parameters^23^. CAIA animals showed induction of clinical symptoms such as periosteal reaction/hypertrophy (PR), bone erosion (B), soft tissue swelling (SS), narrowed joint space (JS) and osteoporosis (OP) in the knee- and ankle-joints (Fig. 3A–D). These were found to be ameliorated with the ASHW, and MTX treatments. The CAIA animals showed high radiological scores in the ankle region (p-value ≤ 0.05) (Fig. 3E). Treatment of the CAIA animals with ASHW (p-value ≤ 0.05) or MTX (p-value ≤ 0.01) showed a significant reduction in the radiological score in the ankle-joint (Fig. 3E). Standard of care drug, MTX showed higher efficacy compared to ASHW in reducing radiological scores in the ankle-joint of the CAIA animals (Fig. 3E insert).

Similarly, knee-joint also showed significant lesions development in the CAIA animals, with increased radiological scores, as compared to the NC animals. Treatment of the CAIA animals with ASHW showed a minor decrease in the radiological scores. However, MTX treatment significantly reduced (p-value ≤ 0.01) radiological scores, as compared to the DC animals (Fig. 3F). Similar to the ankle-joint, MTX treatment in the CAIA animals exhibited higher efficacy as compared to the ASHW in controlling the development of CAIA driven radiological changes in the knee-joints (Fig. 3F insert).

**Figure 4:**
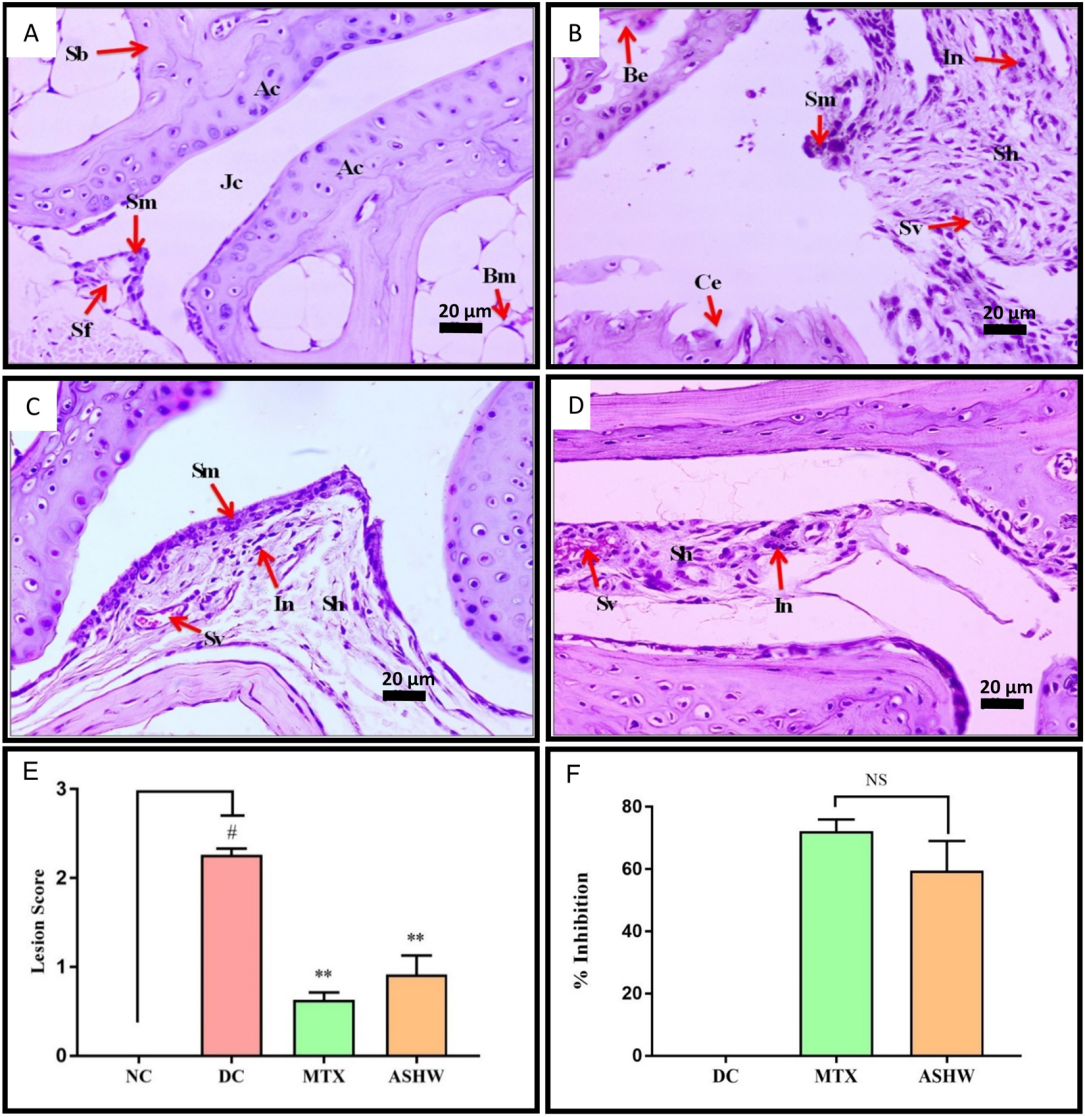
Histopathological Analysis of Ankle Joint. (**A**) Normal control animal ankle-joint parts representing articular cartilage (Ac), synovial membrane (Sm), synovial folds (Sm), spongy Bone (Sb), bone marrow cells (Bm), joint cavity (Jc). (**B**) disease control (DC) animal following treatment C-Ab + LPS showing moderately enlarged synovial membrane (Sm), hyperplastic synovium (Sh), increased synovial vascularity (Sv), inflammation (In), bone erosion (Be), and cartilage erosion (Ce). (**C**) Treatment of CAIA animal with Ashwashila (ASHW) showed mildly enlarged Sm, Sh and increased Sv. (**D**) Diseased animals treated with Methotrexate (MTX) showed minimal enlarged Sh, In and increased Sv. (**E**) Total lesion score measurement indicated increase in inflammatory lesion in the DC animals and reduction following treatment of the animals with ASHW and MTX. (**F**) Similar efficacy of ASHW and MTX in reducing lesion score in the DC animal as a function of percentage (%) inhibition was determined. Values in the results are Mean ± SEM. A one-way analysis of variance (ANOVA) followed by Dunnett’s multiple comparison t-test was used to calculate the statistical difference. Student unpaired t-test was used to calculate the statistical difference in comparison to MTX (p-value # ≤ 0.05; ** ≤ 0.01).

**Figure 5:**
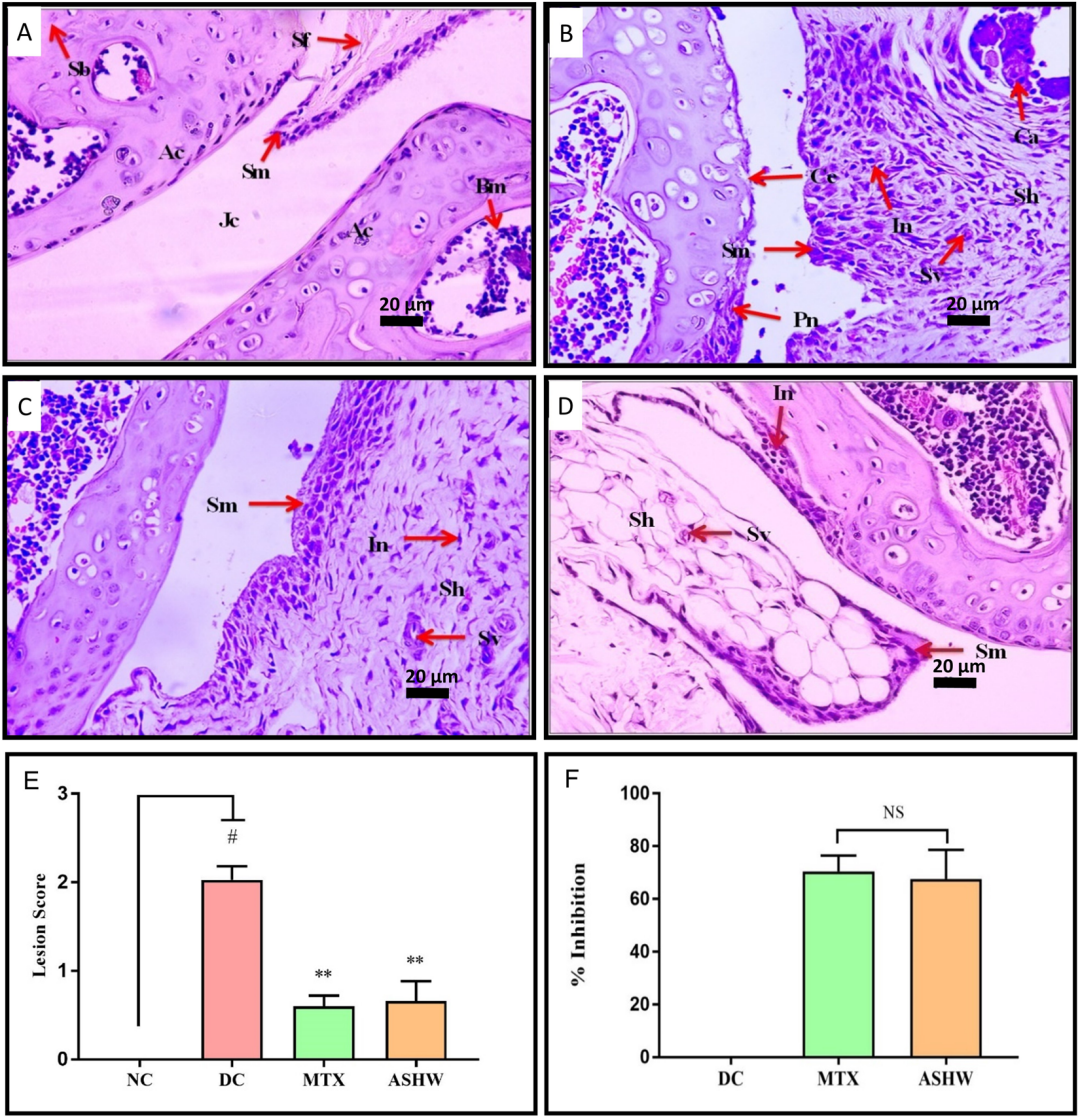
Histopathological Analysis of Knee Joint. (**A**) Normal control (NC) animal knee- joint parts representing articular cartilage (Ac), synovial membrane (Sm), synovial folds (Sm), spongy bone (Sb), bone marrow cells (Bm), joint cavity (Jc). (**B**) Knee-joint in disease control (DC) animal treated with C-Ab + LPS showing moderately enlarged synovial membrane (Sm), hyperplastic synovium (Sh), increased synovial vascularity (Sv), calcinosis (Ca), inflammation (In), pannus formation (Pn) and cartilage erosion (Ce). (**C**) Treatment of the CAIA animal with Ashwashila (ASHW) showed mildly enlarged Sm, Sh, increased Sv, and inflammation (In). (**D**) Treatment of the diseased animal with Methotrexate (MTX) showed mildly enlarged Sm, Sh, increased Sv and In. (**E**) Total lesion score measurement indicated increased inflammatory lesion in the DC animals. Treatment of the diseased animal with ASHW or MTX showed a significant reduction in the lesion score of knee-joints. (**F**) Anti-arthritic efficacy of ASHW and MTX as percentage (%) inhibition showed similar inhibitory effects. Values in the results are Mean ± SEM. A one-way analysis of variance (ANOVA) followed by Dunnett’s multiple comparison t-test was used to calculate the statistical difference. Student unpaired t-test was used to calculate the statistical difference in comparison to MTX (p-value # ≤ 0.05; ** ≤ 0.01).

Histopathological analysis of the CAIA mice pedal was performed following fourteen days of treatment of ASHW and MTX (Figs 4 and 5). Ankle-joints analysis of the DC animals indicated the development of RA disease-associated lesions such as the moderately enlarged synovial membrane, hyperplastic synovium, increased synovial vascularity, the presence of inflammation and, bone and cartilage erosion (Fig. 4). Severe ankle-joint damages in the DC animals were observed through lesion score analysis for synovial-lining cell layer-hyperplasia, -vascularity, infiltration of inflammatory cells, pannus formation, cartilage, and bone erosion (Fig. 4E and Suppl. Fig. 2A–G). Significant reduction in the ankle-joint lesion scores (p-value ≤ 0.01) was observed in the CAIA animals following treatment with ASHW and MTX. Total and individual scoring showed that both the ASHW and MTX exhibited comparable lesion-reducing efficacy in the synovial membrane inflammation, pannus formation, cartilage, and bone erosions of the CAIA animals (Fig. 4F, Suppl. Fig. 2A–G).

Similarly, histopathological analysis of the CAIA animals knee-joints showed a significant increase (p-value ≤ 0.01) in the lesion scores (Fig. 5E). Treatment of the CAIA animals with ASHW and MTX significantly reduced the RA-associated lesions (p-value ≤ 0.01). Individual lesion score analysis in the CAIA animals following ASHW or MTX treatments indicated an equal reduction in synovial membrane inflammation and vascularity along with pannus formation, cartilage and bone erosion (Suppl. Fig. 3A–G). Comparative analysis of the drug efficacy of the MTX and ASHW indicated statistically comparable disease inhibition potentials (Fig. 5F).

**Figure 6:**
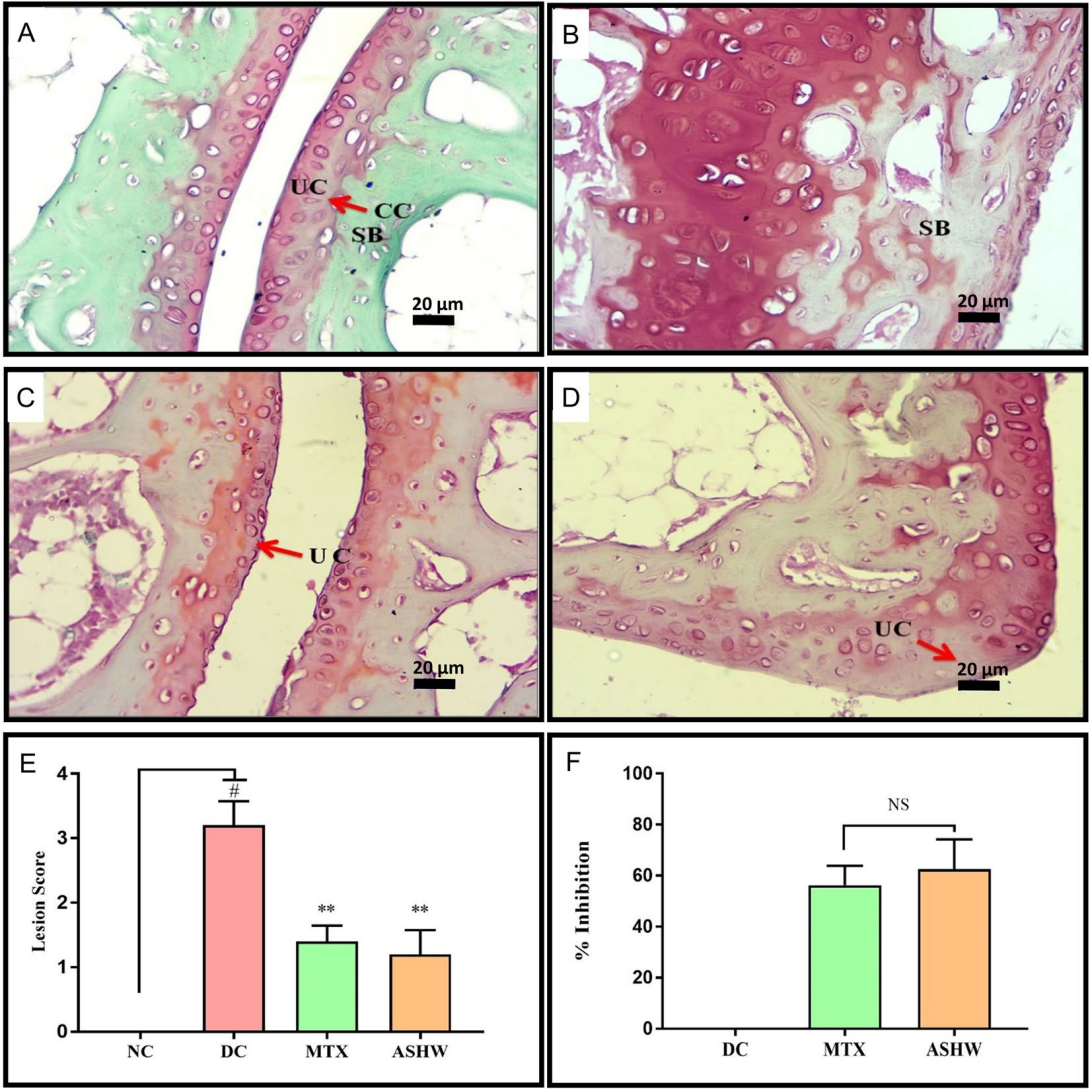
Effect of Ashwashila Treatment on Articular Cartilage Erosion of Ankle Joint. **(A**) Histological analysis of normal control (NC) animal ankle-joint stained with safranin ‘O’ show normal uncalcified cartilage (UC), calcified cartilage (CC), and subchondral bone (SB). (**B**) Ankle joint in disease control (DC) animal following treatment with C-Ab + LPS showed cartilage degradation extending up to SB. (**C**) Treatment of the diseased animal with Ashwashila (ASHW) limited the cartilage degradation till the UC region of the ankle-joint. (**D)** Following treatment of the diseased animals with Methotrexate (MTX) cartilage degradation was limited to UC. (**E**) Inflammatory lesion development was detected in the DC animals that showed significant reduction following treatment of the animals with ASHW or MTX. (**F**) Similar efficacy of ASHW and MTX was observed in anti-arthritic activity through reduction in lesion score as a function of percentage (%) inhibition. Values in the results are Mean ± SEM. A one-way analysis of variance (ANOVA) followed by Dunnett’s multiple comparison t-test was used to calculate the statistical difference. Student unpaired t-test was used to calculate the statistical difference in comparison to MTX (p-value # ≤ 0.05; ** ≤ 0.01).

**Figure 7:**
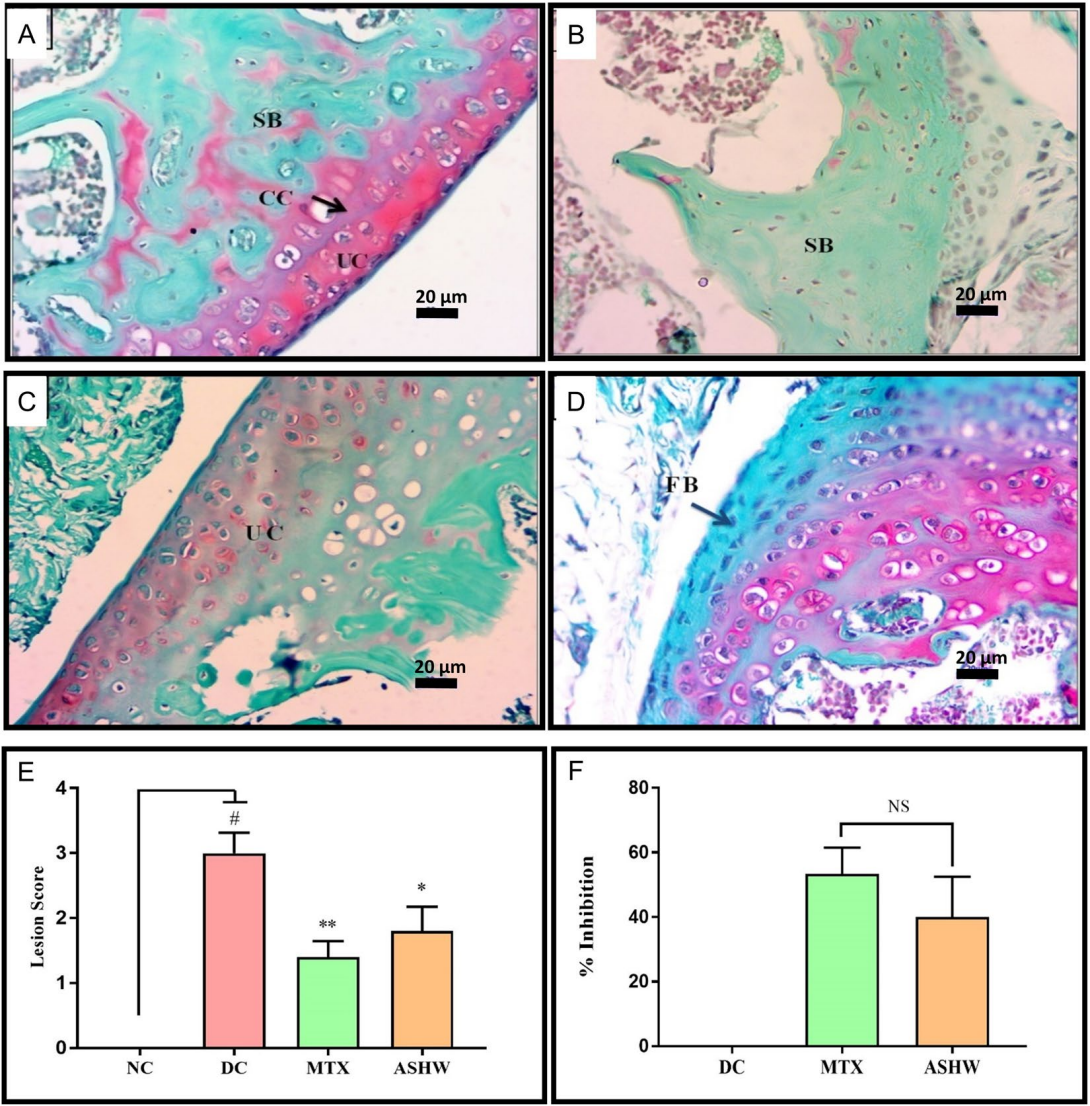
Effect of Ashwashila Treatment on Articular Cartilage Erosion of Knee Joint. (**A**) Histological analysis of normal control (NC) animal knee-joint stained with safranin ‘O’ show normal uncalcified cartilage (UC), calcified cartilage (CC), and subchondral bone (SB). (**B**) Knee-joint in disease control (DC) animal following treatment with C-Ab + LPS showed cartilage degradation extending up to SB. (**C**) Treatment of the diseased animal with Ashwashila (ASHW) limited the cartilage degradation till the UC region of the knee-joint. (**D**) Treatment of the diseased animal with methotrexate (MTX) showed superficial fibrillation of the articular cartilage (FB) region. (**E**) Increase in the pro-inflammatory lesion score was determined in the DC animals that showed reduction following treatment of the animals with ASHW or MTX. (**F**) Similar efficacy of ASHW and MTX in performing anti-arthritic activity was determined through a reduction in lesion score in the treated animals represented as percentage (%) inhibition. Values in the results are Mean ± SEM. A one-way analysis of variance (ANOVA) followed by Dunnett’s multiple comparison t-test was used to calculate the statistical difference. Student unpaired t-test was used to calculate statistical difference in comparison to MTX (p-value # ≤ 0.05; * ≤ 0.05; ** ≤ 0.01).

Stimulation of RA in the CAIA animals induced severe damage and lesion formation in the articular cartilage of the ankle- and knee-joints, as well (Figs 6 and 7). Safranin ‘o’ staining of the proteoglycans present in the cartilage region, showed severe induction of damage to the articular cartilage (p-value ≤ 0.01) in the ankle joints of the DC animals (Fig. 6A,B,E). Treatment of the CAIA animals with ASHW and MTX showed a prominent decrease (p-value ≤ 0.05) in their mean lesion score (Fig. 6C–E). Both ASHW and MTX showed similar efficacy for reducing cartilage damage and lesion formations in the treated CAIA animals. Similar trends were observed in knee joints cartilage analysis. Treatments of the CAIA animal with ASHW and MTX significantly reduced (ASHW: p-value ≤ 0.05; MTX: p-value ≤ 0.01) the disease driven cartilage damages and the formation of lesions over two weeks (Fig. 7A–E). Finally, both the ASHW and MTX showed similar efficacies in modulating disease induced cartilage lesions in the knee-joint (Fig. 7F).

**Figure 8:**
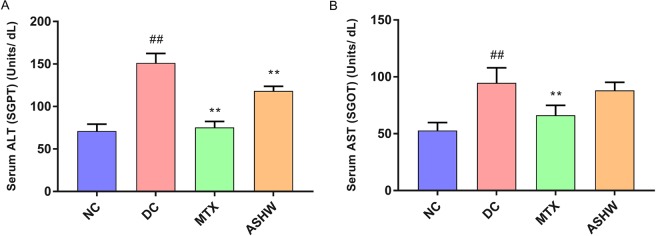
Liver Health Biomarker Measurement in Blood Serum. (**A**) Analysis of the liver enzyme Alanine Aminotransferase (ALT), also known as serum glutamate-pyruvate transaminase (SGPT) was done in the blood serum of the mice showed an increase in the disease control animals (DC) indicating the onset of liver damage as compared to the normal control animals (NC). Treatment of the diseased animals with Methotrexate (MTX) and Ashwashila (ASHW) showed a significant reduction in the levels of ALT post-treatment. (**B**) Analysis of the Aspartate Aminotransferase (AST) also known as serum glutamic-oxaloacetic transaminase (SGOT) enzyme showed a substantial increase in the disease control animals as compared to the healthy control animals. MTX Treatment showed a considerable decrease in the liver toxicity as compared to the DC animals; ASHW treatment showed a minor reduction in the AST levels as compared to the DC animals. Values in the results are Mean ± SEM. A one-way analysis of variance (ANOVA) followed by Dunnett’s multiple comparison t-test was used to calculate the statistical difference. Student unpaired t-test was used to calculate the statistical difference in comparison to MTX (p-value ## and ** ≤ 0.01).

Basic liver functions were studied in the serum of the CAIA mice by analyzing enzymatic biomarkers: Alanine Aminotransferase (ALT) and Aspartate Aminotransferase (AST) (Figs 1 and 8). Balb/c mice treated with C-Ab antibody cocktail and LPS showed a significantly high stimulated release of both ALT and AST (p-value ≤ 0.01) (Fig. 8A,B). Treatment of the CAIA animal with ASHW significantly reduced the ALT back to basal levels (Fig. 8A), while no statistically significant change was observed for the AST biomarker for liver function (Fig. 8B). MTX treatment of the CAIA animals exhibited a substantial decrease in the release of both the ALT and AST biomarkers in the blood serum (Fig. 8A,B). It is noteworthy that ASHW treatment did not induce any additional elevation of serum ALT and AST levels, in comparison to DC animals; suggesting ASHW did not induce any gross level changes in the liver functions of Balb/c mice.

**Figure 9:**
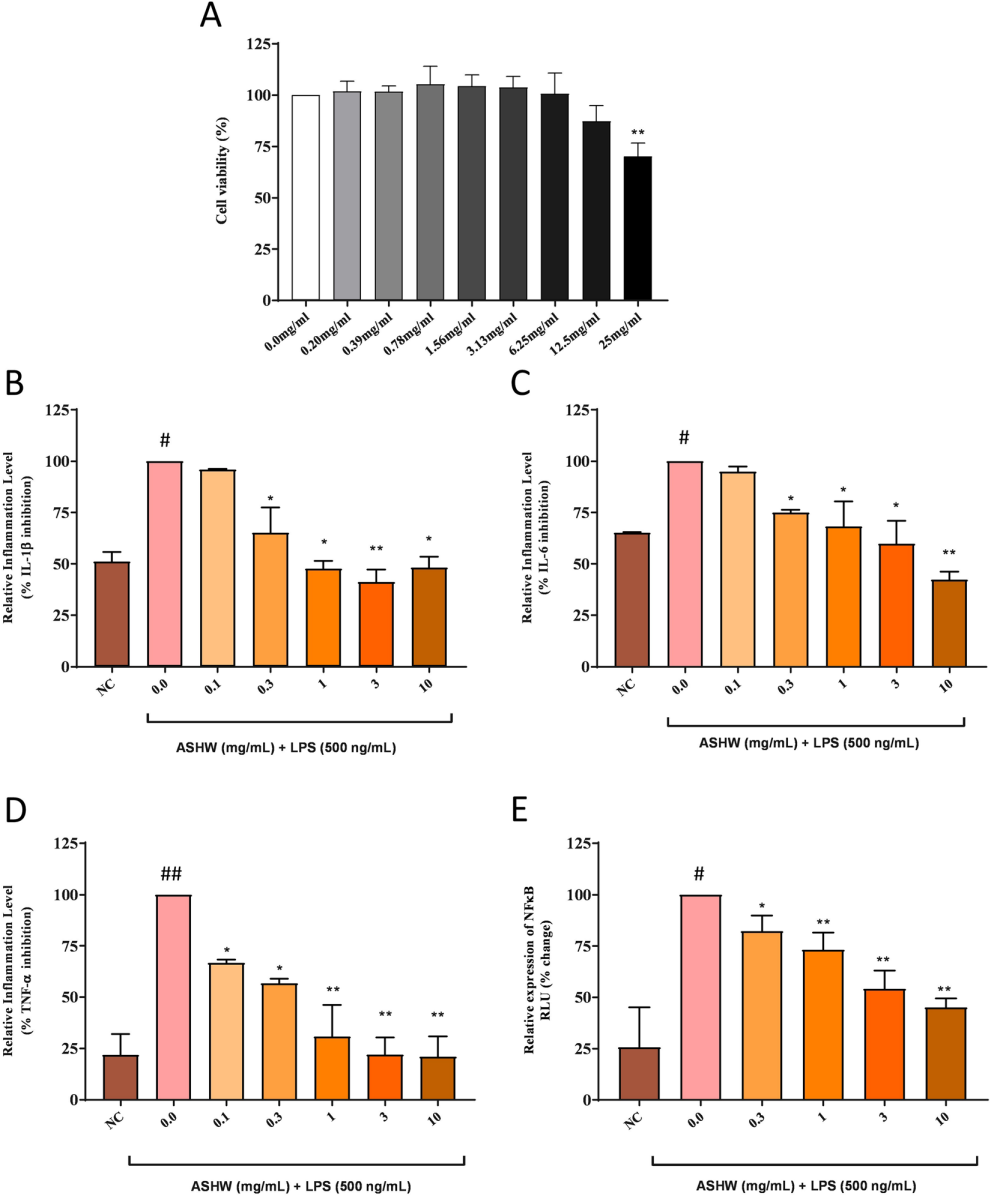
*In-vitro* Modulation of Pro-Inflammatory Cytokines by Ashwashila. (**A**) THP-1 cells treated with varying concentration of the Ashwashila (ASHW) between 0–25 mg/mL induced minor toxicity at dose of ≥12.5 mg/mL. ASHW concentrations to cause 20% and 50% inhibitions were found at 18.83 mg/mL and 42.29 mg/mL, respectively. Pro-inflammatory responses in the endotoxin lipopolysaccharide (LPS) treated THP-1 cells showed stimulated release of the pro-inflammatory cytokines (**B**) IL-1β, (**C**) IL-6 and (**D**) TNF-α. Treatment of the THP-1 cells with varying concentrations of the Ashwashila (ASHW) inhibited the production of the pro-inflammatory cytokines in a dose-dependent manner. (**E**) Luciferase NFκB reporter gene vector transfected THP-1 cells were found to express high quantity of NFκB proteins, when stimulated with LPS. This was reduced in a dose-dependent manner in the cells treated with ASHW up to the tested concentration of 10 mg/mL. Values in the results are Mean ± SEM. A one-way analysis of variance (ANOVA) followed by Dunnett’s multiple comparison t-test was used to calculate the statistical difference. Student unpaired t-test was used to calculate statistical difference in comparison to MTX (p-value # ≤ 0.01; ## ≤ 0.001; * ≤ 0.01; ** ≤ 0.001).

Cell viability analysis of the ASHW in the THP-1 cells showed no loss of cell viability up to 12.5 mg/mL (Fig. 9A). Based on the obtained results, ASHW concentration to induce a 20% loss in cell viability (IC_20_) was calculated at 18.83 mg/mL and IC_50_ at 42.29 mg/mL (Fig. 9A). Statistically significant mild toxicity was detected at the ASHW concentration of 25 mg/mL (p-value ≤ 0.01) (Fig. 9A). It is worthwhile to note that MTX is highly cytotoxic, with reported IC_50_ in the range of 30 μM (equivalent to 13.6 μg/mL), across several cell lines^24^. For elucidating the mechanism of action of the ASHW under *in-vitro* conditions, we studied the modulation of IL-1β, IL-6 and TNF-α cytokines in LPS stimulated human monocytic (THP-1) cells (Fig. 9B–D). All the pro-inflammatory cytokines were found to be upregulated in the THP-1 cells on stimulation with 500 ng/mL LPS. Treatment of the LPS stimulated cells with the ASHW between the concentration of 0.3–10 mg/mL significantly reduced the released levels of IL-1β, IL-6, and TNF-α cytokines in a dose-dependent manner (Fig. 9B). The highest reduction of IL-6 and TNF-α cytokines release in the LPS stimulated THP-1 cells were detected at the ASHW dose of 10 mg/mL (p-value ≤ 0.001) (Fig. 9C,D).

Modulation of the pro-inflammatory upstream gene regulatory protein, nuclear factor kappa-light-chain-enhancer of activated B cells (NFκB), were studied using luminescent reporter gene assay for NFκB in THP-1 cells. LPS stimulation of the THP-1 cells induced a 3-fold increase in the expression of the NFκB protein (Fig. 9E). Treatment of the LPS stimulated THP-1 cells with ASHW significantly reduce the upregulated production of NFκB protein in a dose-dependent manner. Highest inhibition of NFκB expression by ASHW was found at the concentration of 10 mg/mL (Fig. 9E). Taken together, these *in-vitro* results complement well with the *in-vivo* study findings; and supplement the indication that ASHW is indeed a strong anti-inflammatory herbo-mineral formulation.

## Discussion

Tradition Indian Medicines (TIM) have been widely accepted in the public domain as an excellent alternative or additive therapeutics^25^. Disease-modifying anti-rheumatic drugs and non-steroidal anti-inflammatory medicines have been used as the principal therapy for controlling the clinical symptoms associated with RA^26^. Compared to synthetic medicines; herbal formulations are considered to be rather holistic and safe^27^. However, there are limited scientific studies performed on the pre-clinical efficacy of these TIMs in curing chronic and acute diseases. RA is a systemic inflammatory disease that induces inflammation, hyperplasia, auto-antibody production, cartilage and bone destruction, causing pain and immobility in the patients. Ashwashila (ASHW) has been broadly prescribed for the treatment of inflammation, neuropathy, strengthening the physiological and immune system by the traditional Ayurvedic practitioners.

In the present study, we determined the anti-arthritic efficacy of ASHW using collage antibody (C-Ab) induced arthritis (CAIA) Balb/c mice models. The mice dosage of ASHW selected in the study was 353 mg/kg/day (human equivalent dose of 2000 mg/day) given for two weeks; and the standard of care drug, MTX dosage was 0.38 mg/kg given every alternate day for two weeks. Our results showed that ASHW did not modulate the loss of weight, feeding, and water intake habit of the diseased animals, as compared to the MTX. However, both ASHW and MTX showed similar efficacy in reducing the arthritis score, paw and ankle edema, inflammatory lesions in the ankle and knee joints, and pain sensitivity in the CAIA animals. The mode of action for the MTX is well studied, such as, by reducing T-cell activity at the site of inflammation, blocking IL-1β surface receptors of target cells and reducing bone and cartilage damages through erosions^28,29^. However, no information is available regarding the mode of action of ASHW herbal formulation in reducing RA symptoms.

ASHW herbal formulation is composed of an equal quantity of Ashwagandha aqueous extract and Shilajit. Ashwagandha or *Withania somnifera* has been extensively studied for its chemical composition, and its biologically active components identified are alkaloids, steroidal lactones, saponins containing additional acyl group and withanolides^30^. Shilajit is composed of three oxygenated biphenyls and three oxygenated 3-4-benzcoumarins, several phenolics and amino acids and triterpenes^31^. Rasool and Varalakshmi (2007) studied the efficacy of root powder from *Withania somnifera* in modulating the inflammation, oxidative stress and cartilage erosion in adjuvant-induced arthritis in Wistar rat models^32^. The authors showed that the *Withania somnifera* root powder at the daily dose of 1000 mg/kg/day significantly reduced inflammation in the form of lipid peroxidation; and anti-oxidant levels returned to normal levels, as compared to the disease control animals^33^. Other studies have shown the efficacy of *Withania somnifera* extract at the concentrations between 100–800 mg/kg to reduce RA symptoms such as oxidative stress, ankle swelling, lipid peroxidation, glutathione levels, arthritic and radiological score and arthritic pain in the collagen-induced arthritis rat models^9,33^. Shilajit at the concentration of 50 mg/kg/day dose given for 14 days reduced pedal edema by 77% in the carrageenan-stimulated Wistar rats showing inflammation^31^.

In our study, reduction in arthritis score, along with paw, ankle and knee-joint inflammation, and edema in the CAIA animals was observed following a dose of 353 mg/kg/day oral dose of ASHW. Interestingly, since ASHW contains 1:1 ratio by weight of *Withania somnifera* and Shilajit, reduction in RA symptoms such as paw and ankle edema, arthritic and radiological scores were achieved at the individual daily doses of 176.5 mg/kg/day as compared to the other studies, suggesting a synergistic mechanism of action for both of the constituents. Furthermore, ASHW was observed to reduce liver toxicity and damages in the CAIA mice through a reduction in the stimulated levels of Alanine Aminotransferase (ALT) and Aspartate Aminotransferase (AST), in blood serum. This in line with several other studies that have reported the role of *Withania somnifera* extracts in improving liver functions and in ameliorating chemicals induced hepatotoxicity^34–36^.

Using the *in-vitro* cell-based studies, we were further able to elucidate that ASHW was not only biocompatible in the human monocytic (THP-1 cells) but also capable of reducing inflammatory reactions in the LPS-stimulated cells. The inhibitory concentration at 50% loss of cell viability (IC_50_) for ASHW was extrapolated at 42.29 mg/mL. Compared to the RA standard of care drug MTX (IC_50_: 13.6 μg/mL)^24^, the IC_50_ value for ASHW was found to be many order of magnitudes higher; indicating a better safety margin for ASHW.

Studies have demonstrated that pro-inflammatory cytokines play a major role in the severity of RA disease and their reduction also reduces the RA pathogenicity^37,38^. Withanolides present in the *Withania somnifera* extracts are responsible for their anti-inflammatory properties, and in the Shilajit, their phenolic components are accountable for their anti-oxidant and anti-arthritic activities^39,40^. Treatment of human subjects having RA with 5 grams twice daily dose of *Withania somnifera* powder followed by a mineral formulation (sidha makardhwaj) treatment significantly reduced the clinical RA factor; and reduced scores of tender joint counts, swollen joint counts, physician assessment score and pain^41^. Therefore, modulation of the pro-inflammatory cytokines along with attenuation of RA pathogenicity in the CAIA animals by ASHW indicate a direct relationship between the two events. This research paper is the first report on the combined behavior of *Withania somnifera* and Shilajit, in the form of ASHW, in modulating the RA symptoms in diseased animals as there are no scientific reports available on the combined effect of *Withania somnifera* and Shilajit on reducing CAIA systemic and joint inflammation, edema, articular cartilage erosion, and bone erosion.

Earlier studies have reported a direct correlation between the induction of RA pathogenicity, release of pro-inflammatory cytokines and the stimulated expression and release of nuclear factor kappa-light-chain-enhancer of activated B cells (NFκB) in the CAIA animals^42^. Earlier Singh *et al*. (2007), have shown *Withania somnifera* plant crude ethanol extract to reduce LPS stimulated expression of NFκB and associated inflammatory responses^43^. NF-κB protein plays an important role as a mediator for the release of pro-inflammatory cytokines in both innate and adaptive immune cells. In our study, reduction in expression of NFκB protein along with the downstream associated pro-inflammatory cytokines presents the mode of action of ASHW in attenuating RA pathogenicity. Root extracts from *Withania somnifera* have been observed to have chondroprotective effect in cases of other forms of arthritis such as osteoarthritis through the reduction of the nitric oxide in the joints and inhibition of gelatinase activity of the collagenase type 2^44,45^. However, in our study the ASHW treatment did not induce relief from CAIA distress as observed through weight and feeding habit loss of the animals. It is, therefore, possible that the ASHW may require a longer duration of treatment to make significant gross level changes. Observed ASHW driven reversals of bone and cartilage erosion, which is comparable to the MTX treatment, substantiate this hypothesis. The release of various pro-inflammatory soluble mediators and oxidative stress during RA and other systemic inflammations have been implicated in neuroinflammation and cognitive impairments. Both, *Withania somnifera* and Shilajit have been individually observed to possess neuroprotective effect through their anti-oxidant and anti-inflammatory behaviors^8,46^.

Primary afferent neurons perform three major nociceptor functions that are: detection of noxious elements or damaging stimuli (transduction); passage of the resulting sensory input from peripheral terminals to the spinal cord (conduction); and synaptic transfer of this input to neurons within specific laminae of the dorsal horn (transmission)^47^. During severe inflammation such as RA, the threshold for nociceptor neurons to fire action potentials is reduced, leading to pain sensitivity or “hyperalgesia”^48^. This further reduces tissue immune response to damaging stimuli and noxious elements. In the present study, using the Randall-Selitto test and hot plate test we could display that the CAIA animals showed higher pain sensitivity and movement. An oral dose of ASHW helped in the reduction of mechanical and thermal hyperalgesia similar to those observed using the reference drug MTX. This indicated that the ASHW has neuroprotective potentials, as expected.

Taken together, we can conclude that ASHW is capable of reducing RA associated inflammation, oxidative stress and symptoms through modulating the amount of pro-inflammatory mediators. While synthetic drugs may produce rapid relief from RA associated edema and pain, their long term usage and effects on health are always a concern. Herbal formulations, on the other hand, may have milder effects in modulating disease-associated symptoms, but due to their nature derived origin and long-term historical usage, no known side-effects are expected. Therefore, using the CAIA Balb/c mice model, we accentuate that Ashwashila can be a potential candidate for treating rheumatoid arthritis, as a standalone or companion therapy.

## Materials and Methods

### Chemicals and reagents

For the study, Ashwashila (Batch no: AH18/038, manufacturing date: April 2018) was sourced from the Divya Yoga Pharmacy, Haridwar, India, 5-Clone Cocktail antibodies (Cat No-53040) and LPS (Escherichia coli strain 0111: B4; Cat No-9028) were purchased from Chondrex, Inc. WA. Methotrexate (Cat No-M9929) was procured from Sigma Aldrich, St. Louis, MO. Haematoxylin, Potassium Aluminium Sulphate Dodecahydrate, Mercury (II) Oxide red were purchased from Merck India Pvt Ltd, Mumbai, India. Safranin and Fast green were procured from Loba Chemie Pvt. Ltd, Mumbai, India. Eosin Yellow and Ferric Chloride were purchased from Hi-Media Laboratories, Mumbai, India. For tissue culture work, RPMI-1640 cell culture media, Fetal bovine serum, antibiotics, and other reagents were purchased from Life Technologies, Delhi, India.

### Experimental animals

Male Balb/c mice (20–30 g) were procured from Charles River Laboratory licensed supplier, Hylasco Biotechnology Pvt. Ltd, Hyderabad, India. All the animals were housed in polypropylene cages in a controlled room temperature 22 ± 1 °C and relative humidity of 60–70% with 12:12 h light and dark cycle in a registered animal house (Registration Number: 1964/PO/RC/S/17/CPCSEA). The animals were supplied with standard pellet diet (Purina Lab Diet, St. Louis, MO, USA) and sterile filtered water *ad libitum*. The study protocol was approved by the Institutional Animal Ethical Committee of Patanjali Research Institute vide IAEC approval number- PRIAS/LAF/IAEC-009; and all the experiments were performed following relevant guidelines and regulations.

### Induction of arthritis in animals

Arthritis was induced in the Balb/c mice by intraperitoneal (I.P.) injection of a cocktail of 5 monoclonal antibodies to type II collagen (1.5 mg in PBS/mouse; day-0) followed by LPS I.P. injection of 50 μg of lipopolysaccharide (LPS from Escherichia coli strain 011B4; in a sterile normal saline) on day-3 (Fig. 1A). The normal control (NC) animals received an equal volume of PBS along with vehicle Na-CMC. On day-4, disease induced animals were selected and randomized into different groups for treatments:

*Group I:* NC (PBS+ 0.25% Na-CMC p.o.; every day for two weeks)

*Group II:* Disease Control (C-Ab + 0.25% Na-CMC p.o.; every day for two weeks)

*Group III:* C-Ab+ MTX (0.38 mg/kg p.o.; every alternate day for two weeks)

*Group IV:* C-Ab+ ASHW (353 mg/kg p.o.; every day for two weeks)

The vehicle or MTX or ASHW treatment was initiated from day-4 and continued for the next two weeks.

### Assessment of severity of arthritis

The severity of arthritis in each mouse paw was scored every alternate day in a blinded manner according to the marginally modified method of Khachigian (2006) and Moore *et al*. (2014) on a 0–4 scale as follows: 0 = normal; 1 = mild redness, slight swelling of ankle or wrist; 2 = moderate swelling of ankle or wrist; 3 = severe swelling, including some digits, ankle and foot; 4 = maximally inflamed. The total maximum score for each mouse was 16^6,49^. The anti-arthritic activity (%) was calculated for each animal using the following formula: ((Mean arthritis score of disease control animals − arthritis score of each test animal)/Mean arthritis score of disease control animals) × 100.

### Assessment of body weight, feed and water intake habits

Body weight was measured every alternate day. The change in body weight of each animal was calculated before and after the onset of arthritis and after subsequent treatment. Animal feed and water consumption were recorded daily until the end of the experiment.

### Assessment of inflammatory parameters: Measurement of paw and ankle joint thickness

Mouse paw thickness was measured using digital vernier caliper (Mitutoyo, Japan) on days-0, 2, 4, 8, 12, 16 for paw thickness, and days 0, 2, 5, 9, 13, 17 for ankle thickness. The paw and ankle joint edema were calculated by subtracting 0 h (basal) thickness from the respective thickness post-treatment thickness on each day of the study. The anti-inflammatory activity (%) was calculated for each animal using the following formula: ((Mean edema of disease control animals (mm) − edema of each test animal (mm))/Mean edema of disease control animals (mm)) × 100.

### Assessment of pain behaviours

#### Randall Selitto Pressure Test

The Randall–Selitto pressure test was performed to measure static hyperalgesia in animals according to the modified methods of Randall and Selitto (1957) and Anthony *et al*. (2007)^50,51^. The pain response as paw withdrawal threshold (PWT) to the mechanical stimulus was determined with a Randall and Selitto device on day-0, 2, 6, 10 and 14, after 1 hour of drug treatment. The paw withdrawal threshold was defined as the force applied to the dorsal surface of the hind paw that causes mouse to vocalize or withdraw the paw. A limit of 25 g was set to avoid tissue damage. The average of 3 readings with a gap of 5 min from each mouse was recorded in a blinded manner, by a researcher unknown to the treatment conditions.

#### Hot Plate Test

Thermal hyperalgesia was assessed using a hot plate test as described by the methods of Chagasa *et al*. (2017) and Eddy and Leimbach (1953) with minor modifications^52,53^. All the animals were placed into the perspex cylinder of the hot plate (Ugo Saile, Italy) maintained at 55.0 ± 0.5 °C; and time to discomfort reaction (licking paws or jumping) was recorded as response latency. The hot plate test was performed on a day- 0, 2, 7, 11 and 15, after 1 hour of drug treatment. A maximal cut-off point of 20 sec was considered to avoid any possible accidental paw damage. The average of 3 readings with a gap of 5 min from each mouse was recorded in a blinded manner, by a researcher unknown to the treatment conditions.

### Radiological analysis

X-ray analysis was used to assess the morphology of hind limb swelling. After two weeks of drug treatment, all the animals were humanely sacrificed; left hind limbs were isolated and processed for X-rays images using X-ray device (Siemens Heliophos-D Germany) with a 40 KV exposition 0.01 sec (at Department of Radiology, Patanjali Ayurveda Hospital, Haridwar, India). The radiological analysis was done in a blinded manner by the Veterinary Radiologist. Scoring of the abnormalities such as swelling of the soft tissue around the joints, periosteal hypertrophy, narrowing of the joint space, periarticular osteoporosis, bone erosions, and any other lesions were done on the severity level: 0 = Normal; 1 = Slight; 2 = Moderate and 3 = Severe. Knee and ankle joints were analysed separately^54^. The % activity was calculated using the following formula: ((Mean knee or ankle joint radiological score of disease control animals − Knee or ankle joint radiological Score of each test animal)/Mean knee or ankle joint radiological score of disease control animals) × 100.

### Histopathological examination

The mouse right limb was harvested immediately after humanely sacrificed, fixed in 10% buffered-neutral formalin, decalcified in 10% formic acid for four days, embedded in paraffin, sliced solid sections of 3–5 µm thickness and stained with hematoxylin-eosin for general evaluation and Safranine O dye for specific assessment of cartilage damage. Blinded examination of histological slides was performed by a veterinary pathologist to minimize bias. The severity of microscopic arthritic changes (enlargement in synovial lining cell layer, synovial hyperplasia, synovial vascularity, infiltration of inflammatory cells, pannus formation, cartilage erosion, and bone erosion) were evaluated in hematoxylin and eosin (H & E) stained slides using the following grades: 0 = No abnormality detected; 1 = minimal (<1%); 2 = mild (1–25%); 3 = moderate (26–50%); 4 = marked (51–75%); 5 = severe (76–100%). Distributions of the lesions were recorded as focal, multifocal and diffuse. Similarly, the severity of the cartilage degradation was scored as 0 = no apparent change; 1 = superficial fibrillation of articular cartilage; 2 = defects limited to uncalcified cartilage; 3 = defects extends into calcified cartilage; 4 = exposure of subchondral bone at the articular surface. The scoring of knee and ankle joints were recorded separately. Images of the histological slides (H & E and Safranine O) were captured using Olympus Magnus microscope camera, and processed by Olympus MagVision image analysis software.

### Blood biochemistry

For analysis of the Alanine Aminotransferase (ALT) and Aspartate Aminotransferase (AST), the Balb/c mice blood serum was isolated and stored at −80 °C till further use. Commercially available kits for ALT (AL 8304) and AST (AS 8306) were purchased from Randox, USA and processed on RX Monaco technology platform (Randox, USA), as per manufacturer’s instructions.

### Cell culture for *in-vitro* experiments

Human monocytic (THP-1) cells were obtained from the ATCC authorized cell repository, NCCS, Pune, India. THP-1 cells were cultured in RPMI-1640 containing 10% heat-inactivated fetal bovine serum (FBS), in presence of 100 units/mL concentrations of penicillin/streptomycin, 1 mM sodium pyruvate and 4 mM L-glutamine. Cells were grown at 37 °C in a 5% CO_2_/air environment.

### Cell viability analysis

One gram of the powdered ASHW was suspended in incomplete culture media (RPMI-1640) at 37 °C for two hours. The insoluble part was cleared by high-speed centrifuge at 14000 RPM. The cleared fraction was filtered with 0.2 μm filter and stored at 4 °C till further use. THP-1 cells were plated in a 96 well plate at the concentration of 10,000 cells per well in a 96 well plate. The cells were pre-incubated over night and exposed to the ASHW at the concentrations of 0.0, 0.20, 0.39, 0.78, 1.56, 3.12, 6.25, 12.5 and 25 mg/mL for a period of 24 h. At the end of the time period, the exposure medium was removed and the cells were washed with 100 μL PBS. 100 μL of 0.5 mg/mL 3-(4,5-dimethylthiazol-2-yl)-2,5-diphenyltetrazolium bromide (MTT) was added to each well; and the plates were incubated for 3 hours at 37 °C. At the end of the exposure period, the dye was removed and each well was washed with 100 μL PBS. 100 μL of DMSO was added and the plates were placed on a shaker for 10 minutes. Absorbance of each well was then read using the Envision multiplate reader (PerkinElmer, USA) at λ _ABS_ = 595 nm; and cell viability percentage was calculated.

### Cytokines level measurements

Human monocyte THP-1 cells (5 × 10^5^cells/well) were seeded in 24 well culture plates. To study cytokine modulations, media with different concentrations of ASHW was added to the wells at the concentrations of 0.1, 0.33, 1, 3.3 and 10 mg/mL. After treating cells for an hour, LPS was added at final concentration 500 ng/ml except in control wells. Consumed media or cell supernatants were collected after 24 h to measure different cytokines levels such as TNF-α, IL-1β, and IL-6 using standard ELISA kits (BD Biosciences). ELISA assay was performed according to the manufacturer’s protocol, and plates were read at 450 nm using Envision microplate reader (Perkin Elmer, USA).

### Luciferase reporter NFκB gene assay

THP-1 cells were transiently transfected with luciferase reporter vector with NFκB promoter sequence upstream of the luciferase gene. Transfection was performed following the manufacturer’s instruction in 96 well plates using Lipofectamine 3000 (Invitrogen, USA). Two days after transfection, the experiment was performed as described earlier^55^, with some modifications. Used media was replaced with media containing the test compound and control sample. After 1 hour LPS was added at a concentration of 500 ng/ml, where required and incubated further for 12 hours. D-Luciferin salt (Perkin Elmer, USA) at a final concentration of 150 μg/ml was added to the cells and incubated at 37 °C, protected from light. Relative percentage changes in light emission intensity were measured from each well, using Envision microplate reader (Perkin Elmer, USA), LPS induction alone was measured as 100% activity of the NFκB reporter gene^55^.

### Statistical analysis

The data are expressed as the mean ± standard error of the mean (SEM) for each experiment. Statistical analysis was done using GraphPad Prism version 7.0 software. A one-way analysis of variance (ANOVA) followed by Dunnett’s multiple comparison t-test was used to calculate the statistical difference. Student unpaired t-test was used to calculate the statistical difference in comparison to MTX. Values of p < 0.05 were considered statistically significant.

## Supplementary information

Supplementary Info
