# Supplementary material for: Herbo-mineral formulation ‘Ashwashila’ attenuates rheumatoid arthritis symptoms in collagen-antibody-induced arthritis (CAIA) mice model
Source: Sci Rep. 2019 May 29;9:8025. doi: 10.1038/s41598-019-44485-9 (PMC6541602; doi:10.1038/s41598-019-44485-9)
Supplement: Supplementary file 1 — Supplementary Info [file 41598_2019_44485_MOESM1_ESM.pdf]

Supplementary Figures

Title: Herbo-mineral formulation 'Ashwashila' attenuates rheumatoid arthritis symptoms in collagen-antibody induced arthritis (CAIA) mice model

Authors: Acharya Balakrishna[1,2], Sachin Shridhar Sakat[1], Kheemraj Joshi[1], Sandeep Paudel[1], Deepika Joshi[1], Kamal Joshi[1], Ravikant Ranjan[1], Abhishek Gupta[1], Kunal Bhattacharya[1,3], Anurag Varshney[1,2*]

Affiliation:

1. Drug Discovery and Development Division, Patanjali Research Institute, NH-58, Haridwar-249405, Uttarakhand, India.
2. University of Patanjali, Patanjali Yog Peeth, Roorkee-Haridwar Road, Haridwar – 249 405, Uttarakhand, India
3. Center for Nanotechnology and Nanotoxicology, Harvard T.H. Chan School of Public Health, 665 Huntington Avenue, Boston-02115 MA, United States of America.

Corresponding Author:

* Dr Anurag Varshney, PhD MBA

Vice President

Drug Discovery and Development

Patanjali Research Institute

NH-58, Near Bahadrabad,

Haridwar-249405 (UttraKhand)

India

T: +91 1334-244107 x7458 F: +91 1334 244805

Email: Anurag.varshney@prft.co.in

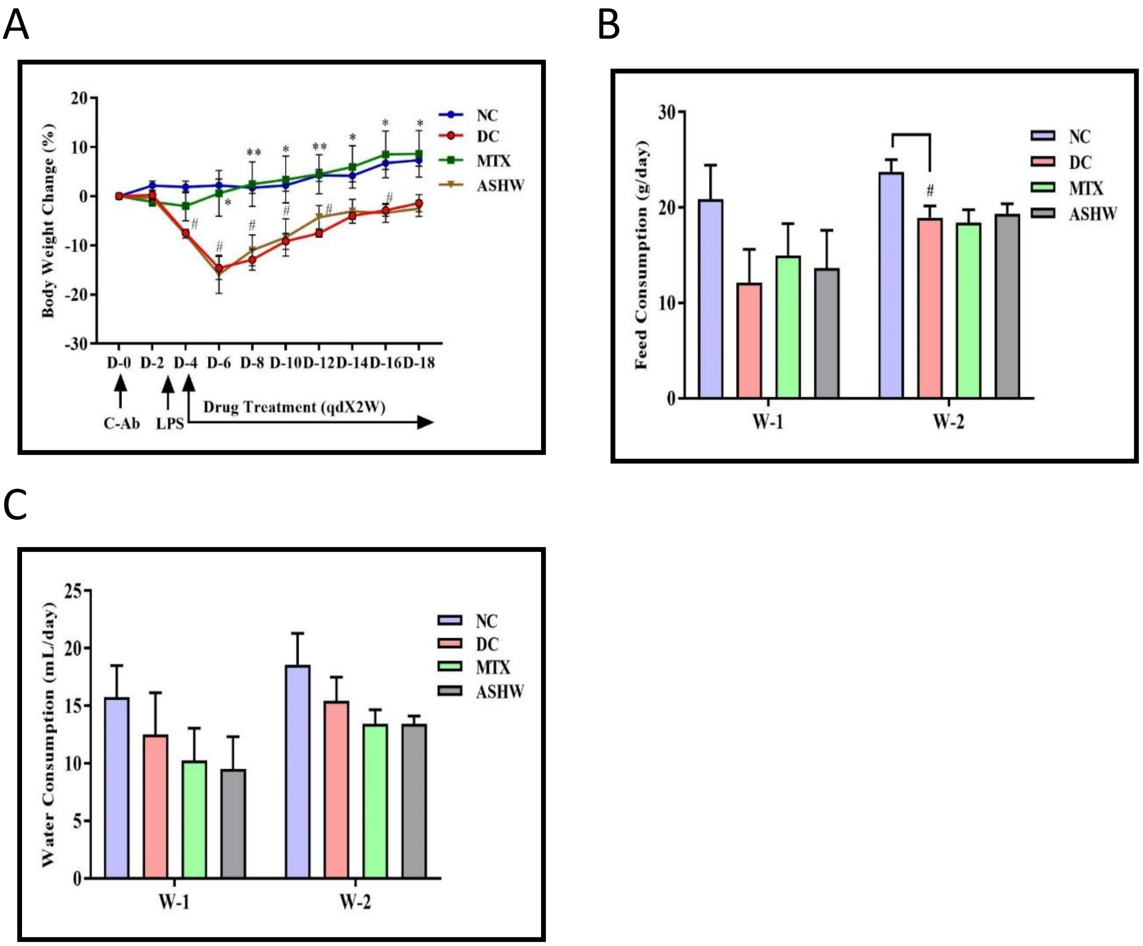

Supplementary Information: Supplementary Figure 1: Body weight and feeding habit change in animal during onset of RA disease. A) Severe loss of body weight was detected in the disease control animals treated with Collagen antibody (C-ab) cocktail and lipopolysaccharide (LPS). No change in the loss of body weight was detected in the Balb/c mice treated with Ashwashila (ASHW). However, normalization of the body weight was observed in the methotrexate (MTX) treated animals; **B)** Diseased animals also lost their feeding habits following the onset of RA disease. No recovery in the feeding habit of the diseased animals was detected following treatment with ASHW or MTX; **C)** Reduced intake of water was detected in the diseased animals following the induction of RA disease. Treatment of the collagen antibody induced arthritis (CAIA) Balb/c mice with ASHW or MTX did not help in the recovery of water intake indicating no change in their distress levels. Values in the results are Mean ± SEM. One-way analysis of variance (ANOVA) followed by Dunnett's multiple comparison t-test was used to calculate statistical difference. Student unpaired t-test was used to calculate statistical difference in comparison to MTX (p value # ≤0.05; * ≤0.05; ** ≤0.01).

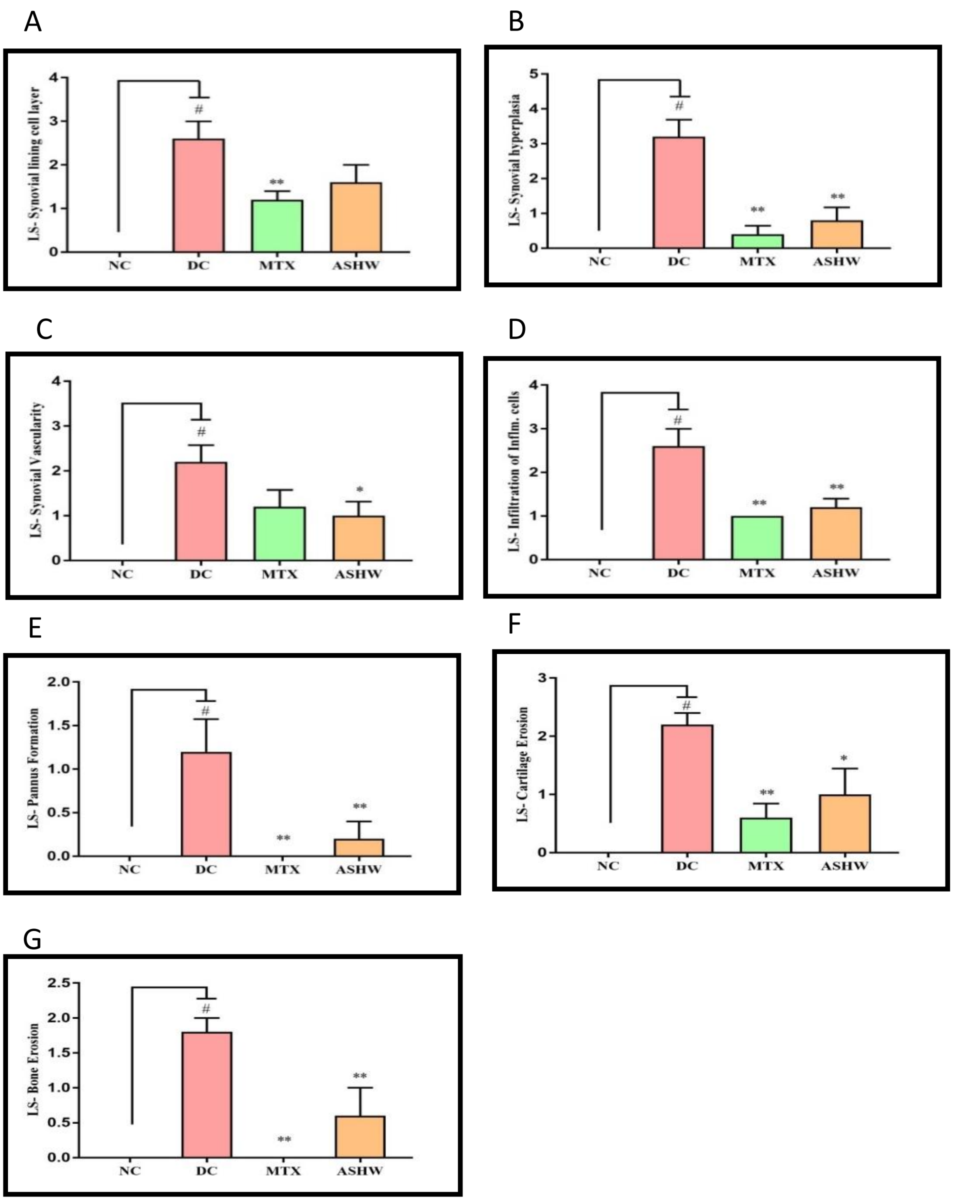

Supplementary Figure 2: Individual lesion scores in H&E stained histopathological samples of ankle-joint region: A) LS-synovial lining cell layer, **B)** LS-synovial hyperplasia, **C)** LS-synovial vascularity, **D)** LS-infiltration of inflammatory cells, **E)** LS-pannus formation, **F)** LS-cartilage erosion, **G)** LS-bone erosion were found to be increased in the disease control Balb/c mice Treatments of the diseased animals with ASHW or MTX significantly reduced all the lesion scores in the knee-joints towards normal levels. Values in the results are Mean ± SEM. One-way analysis of variance (ANOVA) followed by Dunnett's multiple comparison t-test was used to calculate statistical difference. Student unpaired t-test was used to calculate statistical difference in comparison to MTX (p value # ≤0.05; * ≤0.05; ** ≤0.01).

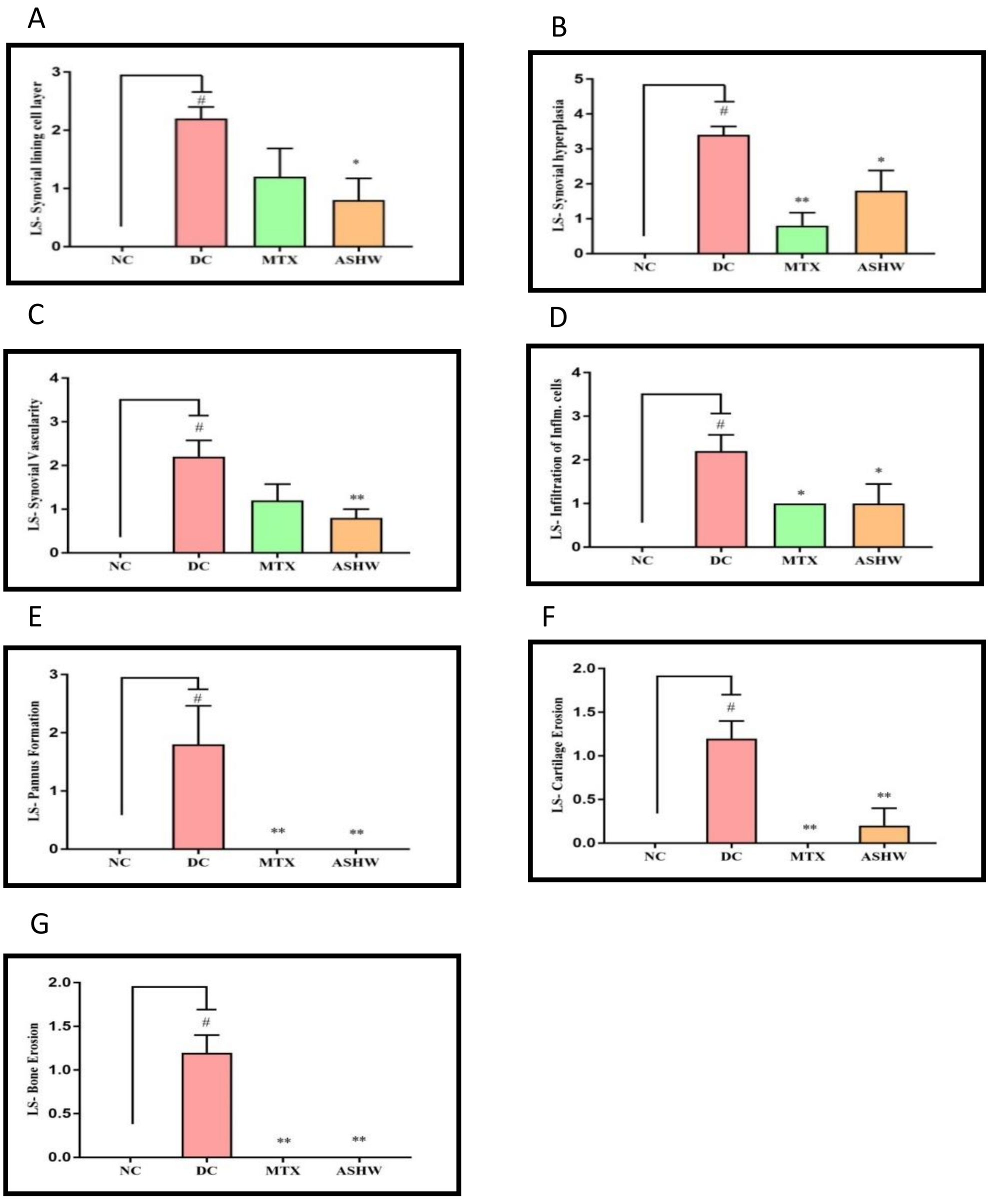

Supplementary Figure 3: Individual lesion scores in H&E stained histopathological samples of knee-joint region: A) LS-synovial lining cell layer, **B)** LS-synovial hyperplasia, **C)** LS-synovial vascularity, **D)** LS-infiltration of inflammatory cells, **E)** LS-pannus formation, **F)** LS-cartilage erosion, **G)** LS-bone erosion were found to be increased in the disease control Balb/c mice. Treatments of the diseased animals with ASHW or MTX significantly reduced all the lesion scores in the knee-joints towards normal levels. Values in the results are Mean ± SEM. One-way analysis of variance (ANOVA) followed by Dunnett's multiple comparison t-test was used to calculate statistical difference. Student unpaired t-test was used to calculate statistical difference in comparison to MTX (p value # ≤0.05; * ≤0.05; ** ≤0.01).
